# Documentation of Drug-Related Problems with ICD-11: Application of the New WHO Code-Set to Clinical Routine Data

**DOI:** 10.3390/jcm12010315

**Published:** 2022-12-31

**Authors:** Wahram Andrikyan, Lea Jung-Poppe, Anna Altenbuchner, Hagen Fabian Nicolaus, Barbara Pfistermeister, Harald Dormann, Martin F. Fromm, Renke Maas

**Affiliations:** 1 Institute of Experimental and Clinical Pharmacology and Toxicology, Friedrich-Alexander-Universität Erlangen-Nürnberg, 91054 Erlangen, Germany; 2 Study Center of the Emergency Department, Fürth Hospital, 90766 Fürth, Germany; 3 Hospital Pharmacy, Fürth Hospital, 90766 Fürth, Germany

**Keywords:** drug-related problem, International Statistical Classification of Diseases and Related Health Problems, ICD-10, ICD-11, medication safety, medication error, adverse drug reaction, ADR, adverse drug event, ADE

## Abstract

Drug-related problems (DRPs), i.e., adverse drug reactions (ADRs) and medication errors (MEs), constitute a serious threat to the patient’s safety. DRPs are often insufficiently captured by clinical routine documentation, and thus, they frequently remain unaddressed. The aim of this study was to assess the coverage and usability of the new 11th revision of the WHO International Classification of Diseases (ICD-11) to document DRPs. We refined the ‘Quality and Safety Algorithm’ from the ICD-11 Reference Guide and used it for DRP reporting to code 100 different anonymized DRPs (50 ADRs and 50 MEs) in a German hospital. The ICD-11 three-part model consisting of harm, cause, and mode was used whenever they were applicable. Of 50 ADRs, 15 (30.0%), such as drug-induced osteoporosis, were fully classifiable and codable by the ICD-11, whereas 35 (70.0%), such as drug-induced hypokalaemia, could not be fully classified due to sanctioning rules preventing the postcoordination (i.e., a combination of specific codes, such as drug and diagnosis). However, coding without the loss of information was possible in the 35 of these 35 (100.0%) ADR cases when we were deviating from the cluster code order of the Reference Guide. In all 50 MEs, the mode could be encoded, but for none of the MEs, postcoordination, i.e., the assignment of the ME to a specific drug, was allowed. In conclusion, the ICD-11 three-part model enables us to acquire more detailed documentation of DRPs than the previous ICD versions did. However, the codability, documentation, and reporting of DRPs could be significantly improved by simple modifications of the current ICD-11 sanctioning rules and by the addition of new ICD-11 codes.

## 1. Introduction

Drug-related problems (DRPs), i.e., adverse drug reactions (ADRs) and medication errors (MEs), are a major cause of patient harm, death, and also cost for the health care system [1,2]. Of special interest are preventable ADRs, which arise from MEs. Globally, the costs of MEs have been estimated to be USD 42 billion annually [1,2]. According to the World Health Organization (WHO), all MEs are potentially avoidable [1]. Therefore, the WHO aimed to reduce the level of severe, avoidable harm related to medications by 50% over 5 years globally, starting from 2017 [1,2].

The international data regarding the prevalence of DRPs show a considerable variation [3]. Differences in the clinical setting, variable definitions of the DRPs, as well as different methods and scrutiny being applied when one is screening for them may explain a large part of the observed variation in reported prevalence of DRPs [4].

However, the coding of DRPs in the routine may also be a key limiting factor for two reasons. The first one is the lack of incentives to document ADRs, and especially MEs, during the clinical routine. The second one is the limitation that commonly used coding systems such as ICD-10 pose for the adequate coding of DRPs. From the perspective of prevention and quality control, it would be important to be able to routinely document and code MEs more consistently than it is currently possible to do with ICD-10.

The precise classification of DRPs using an adequate model is a prerequisite for their reporting (i.e., quality control and research) and prevention [4]. In many clinical settings, the coding of clinical conditions is based on the ICD system. However, the 10th revision of WHO’s International Statistical Classification of Diseases and Related Health Problems (ICD-10) does not provide sufficient coverage of DRPs [5]. For instance, using the ICD-10 system to identify hospital admissions, which are causally related to the administration of a drug, leads to an underestimation of the real number of drug-related hospital admissions by approximately an order of magnitude (0.7% vs. 5–10% according to the literature) [5].

One of the purposes and uses of the current version of ICD (11th revision) is to improve the ability to describe and encode the situation of the patient including the outcome of their treatment and incidents or near-incidents [6]. This improved documentation has the potential to significantly improve patient and medication safety by making DRPs more visible, and thus, more preventable [6].

In this respect ICD-11 allows to conduct postcoordination (i.e., combination/clustering of codes such as the drug and diagnosis involved in an ADR) to provide more specificity in the reporting of clinical routine data [7]. Additionally, a three-part model for coding the causes and mechanisms of healthcare-related adverse events was developed and included in the coding system [8]. Thereby, extension codes enable the assignment of drugs to healthcare-related events. This should allow for more precise coding to be conducted [9].

Following an implicit recommendation from the WHO field trial to assess the ICD-11 framework for classifying patient safety events [10], the aim of this study was to assess the coverage, usability, and limitations of ICD-11 to document DRPs in clinical routine data.

## 2. Materials and Methods

### 2.1. Definitions of Adverse Drug Reaction and Medication Error

In this study, an ADR was defined according to the European Medicines Agency (EMA) as ‘a noxious and unintended response to a medicine’ [11]. This definition does not differentiate between ADRs with and without the contribution of an ME.

An ME is defined according to the EMA as ‘an unintended failure in the drug treatment process that leads to, or has the potential to lead to, harm to the patient’ [12].

### 2.2. Study Design and Origin of Drug-Related Problems

To evaluate the coverage of the ICD-11 code-set, we included 100 different anonymized patient cases with DRPs to be coded using ICD-11. The DRPs were observed in the clinical routine in internal medicine and surgical wards at the large tertiary care Fürth Hospital. The original ADRs were identified, classified, and verified in chart reviews conducted by an interdisciplinary team of clinical pharmacists and pharmacologists, and they were obtained in anonymous form from the routine care data from the ‘medication safety stewardship’ project (German Clinical Trials Register ID 00017534, approval 449_19B of the state accredited Ethics Commission of the Friedrich-Alexander-Universität Erlangen-Nürnberg, which includes a consent waiver for use of anonymized patient data).

The number of ADRs to be assessed was determined in analogy to qualitative interview studies. Information saturation (i.e., data saturation) was assumed to be gained at 50 cases per subgroup [13]. Therefore, 50 different ADRs (22 ADRs resulting from an ME, and 28 ADRs not associated with an ME) as well as additional 50 different MEs that were not (yet) associated with an ADR were selected. The DRPs were documented between August 2020 and August 2021, and they were chosen to represent a broad spectrum of ADRs and MEs with different complexity. All 100 DRPs assessed are shown in Supplementary Table S1 (50 ADRs) and Table S2 (50 MEs).

### 2.3. Coding of the Drug-Related Problems

**Figure 1:**
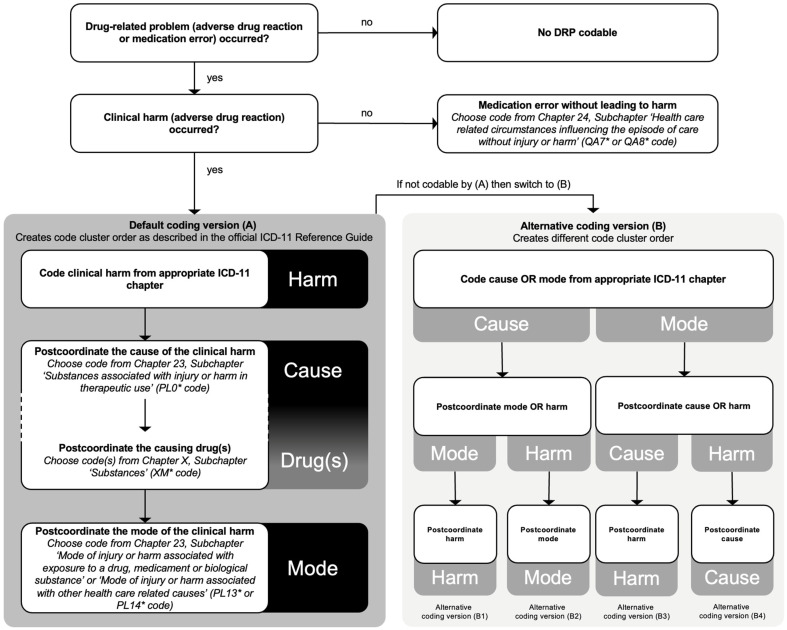
Illustration of the coding process for drug-related problems with ICD-11. This figure is an adaptation (A) and extension (B) of the ‘decision algorithm for when to use the ICD-11 three-part quality and safety model’ from the ICD-11 Reference Guide for drug-related problems [15,16]. Following the default order of coding, (A) creates the code cluster order described in the official ICD-11 Reference Guide. In cases where the default order is not permitted by the official coding tool, the alternative coding orders (B1, B2, B3, and B4) still allow for clinically meaningful coding to be conducted using the tool.

Prior to the analysis, two pharmacists (W.A. and L.J.-P.) with experience in ICD-10 coding completed the ICD-11 training package by the WHO, which consisted of 12 education tool units [14]. We specified the ‘Quality and Safety Algorithm’ from the ICD-11 Reference Guide for DRP coding and used the default coding version (A) for our analysis [15,16] (Figure 1).

The ICD coding of the 100 DRPs was performed using the official coding tool of the WHO in the version of ICD-11 for Mortality and Morbidity Statistics (ICD-11-MMS) 2022-02 [17]. There was an initial agreement of coding on 88% of the cases. When both of the coders coded differently, the case was solved by involving a third reviewer, who is a clinical pharmacologist with ICD-10 coding experience (R.M.).

### 2.4. Cluster Coding with the Three-Part Quality and Safety Model

Whenever it was possible, postcoordination and the three-part quality and safety model were applied for the ICD-11 cluster coding. The WHO says: ‘The three-part model consists of the three components harm (What was the main consequence for the patient’s health?), cause or source of harm (What caused the harm?) and mode or mechanism (In what way? How did the source of harm actually produce harm?)’ [8,15]. The coding process of DRPs with ICD-11 is shown in Figure 1.

### 2.5. Sanctioning Rules

The sanctioning rules, contrary to their name, define the permitted combinations of codes [7,18]. These rules are intended to prevent implausible or impossible code combinations with the aim of increasing the usability of postcoordination systems [7,15,18]. Sanctioning rules are implemented with the official ICD-11 coding tool, which as stated above, was used for the analysis of this study [17]. However, inappropriate definitions or implementations of sanctioning rules can lower the coverage of a coding system. Selected examples with different levels of codability of DRPs and the corresponding ICD-11 clusters are shown in Table 1.

### 2.6. Terminology Mapping with ISO/TR 12300:2014

For the assessment of terminology mapping, a standardized ISO norm was used (ISO/TR 12300:2014) [19]. This norm differentiates between five cases depending on the equivalence of the meaning of the DRP (source term) and the terminology of the ICD-11 system (target term). The equivalence degree scale, which ranges from one to five, with corresponding examples is shown in Table 2.

## 3. Results

### 3.1. Coding of ADRs Using the Three-Part Quality and Safety Model

**Figure 2:**
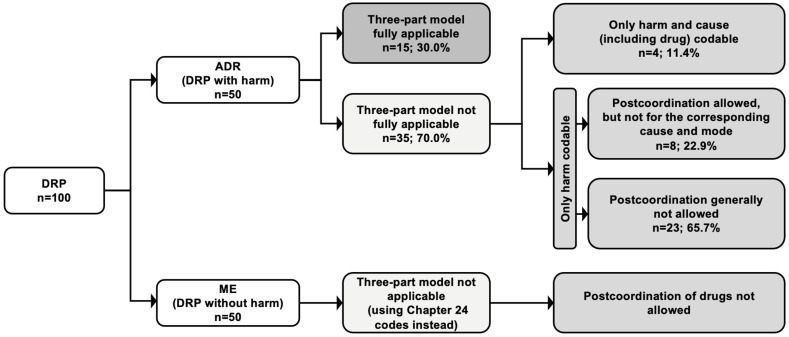
Application of the ICD-11 framework to 100 drug-related problems (50 adverse drug reactions and 50 medication errors without injury or harm). The three-part quality and safety model was applied to events with injury or harm using the coding rules and the default coding order as described in the Reference Guide. For the events without injury or harm, codes from Chapter 24 (Subchapter ‘Health care related circumstances influencing the episode of care without injury or harm’) were used. Abbreviations: ADR, adverse drug reaction; DRP, drug-related problem; ME, medication error.

In total, 15 out of 100 DRPs were completely codable with ICD-11 when we were adhering to the rules and default cluster code order as described in the Reference Guide (Figure 2 and Supplementary Table S1). Only 15 out of 50 (30.0%) ADRs were fully codable using the three-part quality and safety model with the default cluster code order (Figure 1A). For the other 35 ADRs, sanctioning rules prevented the full application of the three-part model. In four cases, the mode could not be added to the cluster, and in eight cases, postcoordination was allowed, but not for coding the corresponding cause and mode. In 23 cases, postcoordination was generally not allowed by the sanctioning rules. For example, it was not allowed to code a gastrointestinal bleeding in more detail as a gastrointestinal bleeding caused by an overdose of warfarin because the sanctioning rules neither allowed the postcoordination of the causing drug (warfarin) nor the mode (overdose) to be combined with the harm (gastrointestinal bleeding).

The ICD-11 codes, for which application of the three-part model was prevented by sanctioning rules, are listed in Table 3.

However, using alternative coding orders (B1, B2, B3 or B4) not mentioned but also not prohibited in the Reference Guide, allowed the full application of the three-part model to all 50 of 50 (100.0%) ADRs.

### 3.2. Terminology Matching of the ADRs with the ICD-11 Codes

As for terminology matching of ADRs regarding the clinical harm, 33 out of 50 (66.0%) showed an equivalence of meaning (equivalence degree scale 1 or 2). In four cases, the documented injury or harm (i.e., source term) was broader and had a less specific meaning than the ICD-11 code did (i.e., target term). These harms were thrombocytopenia, which can only be coded by ‘3B64.12 Drug-induced thrombocytopenic purpura’, leukopenia, which can only be coded using ‘4B00.0Z Neutropenia, unspecified’, and drug-induced anaemia, which can be coded with ‘3A70.10 Drug-induced aplastic anaemia’ (case numbers 3, 14, 39, and 42 in Supplementary Table S1).

In 13 further cases, the originally documented harm was narrower and had a more specific meaning than the ICD-11 code did. For three different harm codes, one ICD-11 code combines two different adverse events, which prevents more specific coding (‘5C72 Hypo-osmolality or hyponatraemia’, ‘5C71 Hyperosmolality or hypernatraemia’, and ‘MA10.0 Elevation of levels of transaminase or lactic acid dehydrogenase’). In the other cases, specific ICD-11 codes were missing (e.g., drug-induced QTc interval prolongation or neuroleptic malignant syndrome) (case numbers 1, 2, 6, 7, 9, 10, 18, 31, 32, 40, 45, 47, and 49).

To code the cause of the ADRs, we always used the code ‘PL00 Drugs, medicaments or biological substances associated with injury or harm in therapeutic use’. The drugs were assigned to the corresponding cause using extension codes from Chapter X. Thereby, we only coded drugs when it was allowed by sanctioning rules. For 32 out of 32 drugs (100.0%), which could be postcoordinated, a corresponding ICD-11 code with an equivalent or synonymous meaning (equivalence degree 1 or 2) was available (Table 4).

A mode could be assigned to the corresponding harm in 15 out of 50 (30.0%) ADRs. Fourteen of these 15 cases showed an equivalence of meaning between the source term (mode) and the ICD-11 code. In one case, the source term (mode) was narrower than the ICD-11 code was. The patient received a medication without indication (case number 15). In this situation, postcoordination was only possible using the code ‘PL13.Y Other specified mode of injury or harm associated with exposure to a drug, medicament or biological substance’.

The detailed analysis and evaluation regarding coverage and terminology matching of ADRs is shown in Supplementary Table S1.

### 3.3. Coding and Terminology Matching of the MEs with the ICD-11 Codes

For MEs without injury or harm, the three-part model does not apply. However, in 41 out of 50 (82.0%), the MEs not associated with clinical harm could be coded by using Chapter 24 codes (Subchapter ‘Health care related circumstances influencing the episode of care without injury or harm’). Nevertheless, for these codes, no postcoordination of the causing drugs was allowed, which made an assignment of the drugs to MEs impossible.

In nine out of 50 cases (18.0%), the ME was narrower and had a more specific meaning than the ICD-11 code did. The MEs without an equivalent ICD-11 code are shown in Table 5. The detailed analysis and evaluation regarding coverage and terminology matching of MEs without injury or harm is shown in Supplementary Table S2.

## 4. Discussion

In this study, we evaluated the applicability and coverage of the ICD-11 code-set to code DRPs in a broad range of DRPs (50 ADRs and 50 MEs without injury or harm) from clinical routine data. Using the coding tool and the coding order as described in the Reference Guide, 15 (30.0%) of the ADRs were fully classifiable and codable by the ICD-11, whereas 35 ADRs (70.0%) were only partly codable due to sanctioning rules preventing the combination of specific codes, such as drug and diagnosis. Only the deviation from the default coding order permitted a clinically meaningful coding of all of the ADRs. Of all 50 MEs, the mode, but not the specific drug(s) involved, could be coded. However, as detailed below, our further analysis of the factors that prevented the correct and detailed coding indicated that making simple adjustments to the current coding rules (i.e., restrictions) could substantially improve the coding of most DRPs, including MEs.

### 4.1. Related Work

In 2010, Stausberg and Hasford assessed the applicability and performance using ICD-10 codes for the identification of adverse drug events (ADEs) [5]. The authors concluded that the ICD-10 system has its limitations because information about medication is not given with ICD-10 codes. This has changed with the introduction of the ICD-11, which allows postcoordination. It should be noted that a simple postcoordination system exists in ICD-10 using the dagger (represented as † or +) and asterisk (represented as *) system. In ICD-10-GM (the German Modification), additional characters exist for flagging a secondary diagnosis and documenting monitoring codes [20]. However, these systems do not reach the coverage and complexity level of ICD-11. Therefore, in a comparative analysis between ICD-10-CM (Clinical Modification) and ICD-11, Fung et al. called postcoordination ‘the potential game changer’ [21].

Our findings fit well into the available data. Forster et al. assessed the applicability of the three-part model of adverse events for 45 patient cases in a WHO field trial [10]. Also, Austin et al. coded 1000 hospital admissions using the three-part model and reported similar difficulties for coding adverse events in the triadic structure [22]. In a larger WHO field trial, Eastwood et al. coded 2896 patient records also using the three-part model when it was applicable and compared it to ICD-10-CA (Canadian Modification) [23]. In all of these studies, the beta drafts of the ICD-11 system were used, and DRPs constituted only a small fraction of the adverse events. Another study from Canada by Chan et al. investigated the application of ICD-11 codes to 573 ADEs, and they pointed out that they had a high coverage, but a poor usability compared to other models [24]. However, the authors did not apply the three-part quality and safety model. Therefore, to the best of our knowledge our work is the first study to assess the ICD-11 codes with a broad variety of DRPs (i.e., ADRs and MEs).

### 4.2. Inter-Rater Reliability Using ICD-11

It is known that inter-rater reliability is not perfect when patient cases are coded via ICD-11 [10,23]. In this study, the initial inter-rater agreement was 88%. During the study, we identified DRPs, which in theory can be interpreted in different ways. For instance, it is not pointed out in the Reference Guide if absolute or relative contraindications should be coded with PL13.7.

A more complicated example would be the prescription of amlodipine 5 mg combined with simvastatin 40 mg. A simvastatin treatment with doses > 20 mg is contraindicated in combination with amlodipine according to the German Summary of Product Characteristics (SmPC) [25]. Should this ME be coded with: (a) a contraindication (PL 13.7), (b) an overdose (PL 13.0), or (c) a drug interaction (PL 13.9)?

Another ambiguous example is the incompatibility of two drugs (e.g., iron and levothyroxine in case number 55). Is this an incorrect administration or a drug interaction? We finally agreed on it being a drug interaction, and we concluded that these cases should be specified in the Reference Guide.

The inter-rater variability of ICD-11 coding may also arise from the classification of a clinical event as an ADR, which usually requires clinical judgment, because alternative causes and other contributing causes have to be considered. Bleeding may be caused by warfarin, but it may also be caused by a platelet deficiency, a trauma, a vascular malformation, or any combination of these. In clinical practice, very few events (such as hypoglycemia during an insulin treatment) can be related to drug prescriptions with a causality rating of ‘certain’.

To overcome this problem, the Reference Guide provides a list with ‘Connecting terms implying a causal relationship’, ‘Connecting terms where the causal relationship is unclear’, and ‘Connecting terms NOT implying a causal relationship’ (Chapter 2.23.20.2 ‘Causation in the context of quality and safety’) [15]. In our opinion, this system is not precise enough. For example, in clinical routine, it is common to document a ‘cough after initiation of ACE inhibitor treatment’ for a suggested causal association between treatment and the event cough, although the Reference Guide recommends that the term ‘after’ implies that there is no causal relationship.

Adding codes of the World Health Organization-Uppsala Monitoring Centre (WHO-UMC) scale as extension codes (such as certain, probable/likely, possible, unlikely, conditional/unclassified, and non-assessable/unclassifiable), which specify the causality of the DRPs could improve the coding system, but this would also add further complexity to the code-set [26].

These examples show that ICD-11 coding is more complex than former ICD versions are. As already pointed out in the WHO field trial, assessing the ICD-11 framework for classifying patient safety events, sufficient training and teaching are necessary [10].

### 4.3. Errors of Omission and Missing Indication

One of the strengths of the ICD-11 code-set is that errors of omission can be mapped using ‘PL14.0 Non-administration of necessary drug’. It is known from the literature that not all the DRP models cover these types of events [4]. However, a medication prescribed without indication is not covered by the ICD-11 system. Therefore, we suggest a new code for this fairly common case (Table 5). This would also allow a more specific coding of case number 15 (osteoporosis due to prednisolone treatment without clinically justifiable indication) and be in concordance to the Pharmaceutical Care Network Europe (PCNE) classification system for DRPs [27].

### 4.4. Suggestions for Potential Improvements of ICD-11 Coding

**Figure 3:**
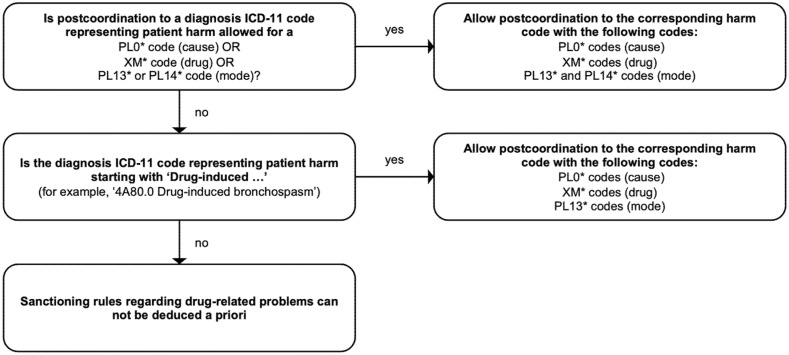
Proposed algorithm for the automated generation/deduction of more precise sanctioning rules regarding drug-related problems. The algorithm checks for two things. On the one hand it asks: Can cause OR drug OR mode be postcoordinated to an ICD-11 code representing patient harm? On the other hand, it asks: Is the ICD-11 code precoordinated and starting with the term ‘Drug-induced…’? Then, allow for the full application of the three-part quality and safety model.

Firstly, sanctioning rules need to be included to the ICD-11 codes listed in Table 3. This would allow for the full application of the three-part quality and safety model to the 35 ADRs and therefore a better coverage of the DRPs. For these codes, we suggest allowing the coding of cause and mode from ICD-11, Chapter 23, Subchapter: ‘Causes of healthcare related harm or injury’. For example, it is not conceivable that the current sanctioning rules do not allow the combination of the very common adverse drug effect hypokalaemia with a drug. Especially for the category of precoordinated codes starting with the term ‘Drug-induced…’, one would expect that coding of the causing drug and the mode (such as overdose) is allowed. In Figure 3, we propose an algorithm with which sanctioning rules for DRPs can be easily deduced a priori and in more detail. The implementation of the algorithm would, amongst other things, allow the full application of the three-part model to cases, in which postcoordination of the cause is allowed, but not for the corresponding mode (Table 3).

For more specific coding of ADRs, we suggest the inclusion of extra codes for drug-induced QT(c) interval prolongation (i.e., drug induced long QT syndrome (LQTS)), drug-induced anaemia, leukopenia, heparin-induced thrombocytopenia type 1 or 2, acutely decompensated chronic heart failure, and neuroleptic malignant syndrome. This list covers some common issues, but it is non-exhaustive. In addition, the ICD-11 codes combining two different clinical conditions should be split into separate codes (e.g., split the codes ‘5C72 Hypo-osmolality or hyponatraemia’, ‘5C71 Hyperosmolality or hypernatraemia’, and ‘MA10.0 Elevation of levels of transaminase or lactic acid dehydrogenase’). Using these ICD-11 terms is always associated with a loss of clinical precision. The finding that ICD codes are often not specific enough to cover clinical conditions is not new, and it has been pointed out by research in the past. For example, in 2008, Molokhia et al. already suggested the inclusion of an ICD code for ‘drug-induced LQTS/TdP’ [28]. In ICD-11, a code for (congenital) LQTS exists. However, with the new postcoordination system, a distinction between primary and secondary (e.g., drug-induced) LQTSs could easily be implemented.

As pointed out in an earlier study, the classification of an ADR as an event involving an ME depends on the context of the event itself [4]. For example, the classification of anaphylaxis caused by amoxicillin may or may not involve an ME depending on whether or not the patient had a known allergy to it. Providing the necessary information and assessing it during the coding process would be too complicated in many cases. Therefore, we suggest an additional (optional) extension code ‘involving medication error’ to flag MEs leading to preventable ADRs, which are of special interest in medication safety and quality management.

The three-part quality and safety model does not apply to MEs without injury or harm. However, these events can be described by the Chapter 24 codes (Subchapter ‘Health care related circumstances influencing the episode of care without injury or harm’) [15]. We suggest the provision of sanctioning rules and codes allowing for the assignment of drugs to MEs not causing patient harm.

Finally, to improve coverage and differentiation of MEs with and without injury or harm, we suggest the addition of codes to Chapters 23 and 24 (Subchapters: ‘Health care related circumstances influencing the episode of care without injury or harm’ and ‘Mode of injury or harm associated with exposure to a drug, medicament or biological substance’, respectively). The suggested changes are shown in Table 5 and would lead to perfect terminology matching for the mode of all the ADRs and also MEs without injury or harm. Further granularity could be achieved by stratification of the drug interactions and contraindications into drug–drug and drug–disease interactions with the postcoordination of the interaction partner. This concept could also be applied to overdose codes via stratification with renal and hepatic impairment or generally allowing the assignment of causing diagnosis codes (e.g., renal impairment causing an overdose of a drug without injury or harm).

To avoid misinterpretation, we would like to point out that it is possible to apply the three-part model to the remaining 35 ADRs when one is starting to code with the cause or mode instead of the harm. This leads to code clusters with a different order than the predetermined order in the ICD-11 Reference Guide (e.g., cause-harm-mode instead of harm-cause-mode, see Figure 1) [15]. All of the different code clusters confer the same information and are mutually interchangeable because the order of causality is not altered as it is possible with other stem code clusters. However, in our opinion the order of ADR coding using the three-part model should always be the same. Otherwise, the duration of the coding process is extended unnecessarily because one would have to figure out the order to code every single case.

To sum up, so far optimal coding of DRPs is prevented by imprecise ICD-11 codes and missing sanctioning rules.

### 4.5. Possible Applications of the New ICD-11 System

ICD-11-MMS was primarily developed for documentation of mortality and morbidity. The underreporting of diagnoses, especially when they are not deemed to be relevant for reimbursement, may be a common issue in the clinical routine, which is a limitation to any framework used to classify adverse events [29]. However, if it is used appropriately, the ICD-11 code-set allows for a broad variety of potential applications compared to older revisions.

One application could be the generation of automated medication safety reports, which was already proposed in principle by Stausberg and Hasford. in 2010 [5]. Using clinical routine data for surveillance and detection of DRPs could help in identifying drugs or drug classes with higher rates of dosing errors, drug interactions, and contraindications. The improved ICD-11 coding of DRPs has the potential to profoundly improve medication safety, as it would enable an automated and thus broader and much faster identification of the safety signals. The current reporting of newly observed ADRs by physicians and pharmacists, albeit mandatory in countries such as Germany, is grossly underused as it is neither reimbursed, nor is non-compliance effectively sanctioned.

Extension codes produce detailed information regarding description and timing of diagnoses [9]. For example, the postcoordination of ‘XY0Y Main condition’ indicates the reason for hospitalization, whereas ‘XY6M Present on admission’ flags chronic conditions. Using this information in an ICD-11 coded dataset, the number of drug-related hospital admissions could be automatically identified. In addition, extension codes indicating the diagnosis time (e.g., ‘XY9N Intraoperative’ or ‘XY7V Postoperative’) could give more insights into potential correlations with preoperative drug prescriptions.

Another application could be a documentation system for pharmaceutical services (e.g., medication analyses for polypharmaceutic patients receiving oral antitumor therapy or immune suppressive medication). For instance, in Germany and China pharmaceutical services are reimbursable since 2022 [30,31]. Here, using ICD-11 to report DRPs may allow for the better capturing and understanding of common pharmaceutical interventions.

### 4.6. Limitations

Even though the 100 different DRPs we assessed can be considered sufficient to reach ‘information saturation’, it is very likely that there are other DRPs which may expose additional limitations or a need for further clarification of the ICD-11 coding. However, one strength of our study is that we used DRPs which were identified, verified, and documented in detail by an interdisciplinary team of medication safety experts. In this respect, the high proportion of currently unresolved but potentially easily resolvable coding issues identified in the present analysis should already be sufficient to motivate efforts aimed at improving the current ICD-11 rules.

## 5. Conclusions

In comparison to former ICD versions, the new ICD-11 system allows for a better coverage of DRPs (i.e., ADRs and MEs) in the clinical routine data. This is mainly attributable to new postcoordination allowed by ICD-11. Switching coding in the clinical routine from ICD-10 to ICD-11 has the potential to improve medication safety research and quality control. However, some coding limitations remain, and the usability and coverage can and should be significantly improved by adding new ICD-11 codes and sanctioning rules.

## Figures and Tables

**Table 1:** Selected examples of the codability with ICD-11 of three different drug-related problems. The first example (ADR) shows a fully codable drug-related problem, whereas for the second case (ADR), the official ICD-11 coding tool allowed no postcoordination when we were following the code cluster order of the Reference Guide. However, it could be coded using an alternative code cluster order. The third case (ME) could not be adequately coded by ICD-11. ICD-11 codes are shown in bold. Abbreviations: DRP, drug-related problem; N.A., not applicable.

DRP	Harm	Cause	Drug(s)	Mode	ICD-11 Code Cluster Order as Described in Reference Guide	Alternative ICD-11 Code Cluster Orders
Osteoporosis due to prednisolone treatment without clinically justifiable indication	**FB83.13** *Drug-induced osteoporosis*	**PL00** *Drugs, medicaments, or biological substances associated with injury or harm in therapeutic use*	**XM6JJ4** *Prednisolone*	**PL13.Y** *Other specified mode of injury or harm associated with exposure to a drug, medicament, or biological substance*	**FB83.13/PL00&XM6JJ4/PL13.Y**	**FB83.13/PL13.Y/PL00&XM6JJ4** **PL00&XM6JJ4/FB83.13/PL13.Y** **PL00&XM6JJ4/PL13.Y/FB83.13** **PL13.Y/FB83.13/PL00&XM6JJ4** **PL13.Y/PL00&XM6JJ4/FB83.13**
Hypokalaemia due to hydrochlorothiazide treatment	**5C77** *Hypokalaemia*	**PL00** *Drugs, medicaments, or biological substances associated with injury or harm in therapeutic use*	**XM6910** *Hydrochlorothiazide*	**PL13.2** *Drug-related injury or harm in the context of correct administration or dosage, as mode of injury or harm*	**5C77** (no postcoordination allowed)	**PL00&XM6910/5C77/PL13.2** **PL00&XM6910/PL13.2/5C77** **PL13.2/5C77/PL00&XM6910** **PL13.2/PL00&XM6910/5C77**
Contraindication: Metformin treatment in combination with GFR < 30 mL/min	N.A.	N.A.	N.A.	**QA77** *Medication or substance that is known to be contraindicated for the patient without injury or harm*	**QA77** (no postcoordination allowed)	N.A.

**Table 2:** ISO/TR 12300:2014 equivalence degree scale. Example cases of the application of the method for terminology mapping (ISO/TR 12300:2014) to drug-related problems.

Equivalence Degree Scale ISO/TR 12300:2014	Definition	Example Mapping Case of Source and Target Terms (i.e., ICD-11 Term)
1	Equivalence of meaning: lexical and conceptual;	Hyperkalaemia and Hyperkalaemia
2	Equivalence of meaning, but with synonym;	Hypocalcaemia and Calcium deficiency
3	Source term is broader and has less specific meaning than target term;	Leukopenia and Neutropenia
4	Source term is narrower and has more specific meaning than target term;	QTc interval prolongation and cardiac arrhythmia
5	No mapping possible. No term was found in the target with some degree of equivalence.	-

**Table 3:** ICD-11 codes describing injury or harm, for which the full application of the three-part quality and safety model to the respective adverse drug reaction was not allowed due to sanctioning rules. This table also provides the information about which sanctioning rules (type of postcoordination codes) should be added (allowed) to allow for the full application of the three-part model.

Harm ICD-11 Code	Meaning	Reason, Which Prevents Full Application of the Three-Part Model
1F23.2	Candidosis of gastrointestinal tract	Postcoordination allowed, but not for the corresponding cause and mode
4B00.0Z	Neutropenia, unspecified	Postcoordincation generally not allowed
5A02.4	Thyrotoxicosis factitia	Postcoordincation generally not allowed
5B5K.1Z	Calcium deficiency, unspecified	Postcoordincation generally not allowed
5C71	Hyperosmolality or hypernatraemia	Postcoordincation generally not allowed
5C72	Hypo-osmolality or hyponatraemia	Postcoordincation generally not allowed
5C76	Hyperkalaemia	Postcoordincation generally not allowed
5C77	Hypokalaemia	Postcoordincation generally not allowed
6C44.20	Sedative, hypnotic or anxiolytic dependence, current use	Postcoordincation for the cause allowed, but not for the corresponding mode
6C4F.5	Delirium induced by multiple specified psychoactive substances including medications	Postcoordincation for the cause allowed, but not for the corresponding mode
8A63.Y	Seizure due to other acute cause	Postcoordincation for the cause allowed, but not for the corresponding mode
BA21	Orthostatic hypotension	Postcoordincation generally not allowed
BD1Z	Heart failure, unspecified	Postcoordination allowed, but not for the corresponding cause and mode
BD4Z	Chronic arterial occlusive disease, unspecified	Postcoordination allowed, but not for the corresponding cause and mode
DB95.Z	Drug-induced or toxic liver disease, unspecified	Postcoordincation for the cause allowed, but not for the corresponding mode
GB60.2	Acute kidney failure, stage 3	Postcoordination allowed, but not for the corresponding cause and mode
MA10.0	Elevation of levels of transaminase or lactic acid dehydrogenase	Postcoordincation generally not allowed
MA10.1	Abnormal levels of other specified serum enzymes	Postcoordincation generally not allowed
MB48.0Y	Other specified vertigo	Postcoordincation generally not allowed
MC81.1	Bradycardia, unspecified	Postcoordincation generally not allowed
MD11.5	Dyspnoea	Postcoordincation generally not allowed
MD90.0	Nausea	Postcoordincation generally not allowed
ME05.0	Constipation	Postcoordincation generally not allowed
ME24.9Z	Gastrointestinal bleeding, unspecified	Postcoordination allowed, but not for the corresponding cause and mode
ME24.A1	Haemorrhage of anus and rectum	Postcoordination allowed, but not for the corresponding cause and mode
ME24.A5	Haematemesis	Postcoordination allowed, but not for the corresponding cause and mode
MF50.3	Retention of urine	Postcoordincation generally not allowed
MG29.00	Ankle oedema	Postcoordincation generally not allowed

**Table 4:** Application of the method for terminology mapping (ISO/TR 12300:2014) to the codable drug-related problems. This table shows the number of codable items with the corresponding categorization into the equivalence degree scale. An item is codable when postcoordination is allowed by the official ICD-11 coding tool. For medication errors without injury or harm postcoordination is generally not allowed so that causing drugs are not codable. Abbreviations: ADR, adverse drug reaction; ME, medication error.

	ADR				ME
Equivalence Degree Scale ISO/TR 12300:2014	Harm (Codable n = 50)	Cause (Codable n = 19)	Drug (Codable n = 32)	Mode (Codable n = 15)	Mode (Codable n = 50)
**1** Equivalence of meaning: lexical and conceptual	20 (40.0%)	0 (0.0%)	31 (96.9%)	0 (0.0%)	0 (0.0%)
**2** Equivalence of meaning, but with synonym	13 (26.0%)	19 (100.0%)	1 (3.1%)	14 (93.3%)	41 (82.0%)
**3** Source term is broader and has less specific meaning than target term	4 (8.0%)	0 (0.0%)	0 (0.0%)	0 (0.0%)	0 (0.0%)
**4** Source term is narrower and has more specific meaning than target term	13 (26.0%)	0 (0.0%)	0 (0.0%)	1 (6.7%)	9 (18.0%)
**5** No mapping possible. No term was found in the target with some degree of equivalence	0 (0.0%)	0 (0.0%)	0 (0.0%)	0 (0.0%)	0 (0.0%)

**Table 5:** Medication errors which could not be coded with sufficient specificity. This table also suggests changes for better coverage of Chapter 23 and 24 codes (Subchapters: ‘Health care related circumstances influencing the episode of care without injury or harm’ and ‘Mode of injury or harm associated with exposure to a drug, medicament or biological substance’, respectively). Abbreviations: ME, medication error.

ME without Injury or Harm	Suggested Change
Halving of tablets although not allowed	Consider additional code for ‘Inappropriate halving of tablets without injury or harm’
Duplicate prescription	Consider additional code for ‘Duplicate prescription without injury or harm’
Prescription with missing indication	Consider additional code for ‘Medication without clinically justifiable indication without injury or harm’
Wrong dosing interval	Consider splitting the code QA74: Unspecified appropriateness of dosing or administration without injury or harm (‘Inappropriateness of dosing without injury or harm’ and ‘Inappropriateness of administration without injury or harm’)
Missing therapeutic drug monitoring	Consider additional code for ‘Clinically required therapeutic drug monitoring omitted or missing without injury or harm’

## Data Availability

The anonymized patient data used in this study are available in Supplementary Tables S1 and S2.

## Supplementary Materials

The following supporting information can be downloaded at: https://www.mdpi.com/article/10.3390/jcm12010315/s1, Table S1: List of drug-related problems (adverse drug reactions) included in the study; Table S2: List of drug-related problems (medication errors) included in the study.
